# Objective quantification of chemotherapy-induced madarosis: a pilot study of an automated computer vision pipeline for eyebrow density assessment

**DOI:** 10.1038/s41598-026-48967-5

**Published:** 2026-04-13

**Authors:** M. González, A. Sahila, A. Rodrigues, P. Bernardo, D. Gonzalez-Aguilera, I. Barbero-García

**Affiliations:** 1 University School of Nursing of Ávila, Universidad de Salamanca, Salamanca, Spain; 2 Department of Cartographic and Terrain Engineering, Higher Polytechnic School of Ávila, Universidad de Salamanca, Salamanca, Spain; 3 Institute of Biomedical Research of Salamanca (IBSAL), University Hospital of Salamanca, Salamanca, Spain; 4 Marqués de Valdecilla University Hospital, Santander, Spain

**Keywords:** Chemotherapy-induced alopecia, Madarosis, Breast cancer, Computer vision, Image analysis, Eyebrow loss prevention, Cancer, Computational biology and bioinformatics, Health care, Medical research, Oncology

## Abstract

Chemotherapy-induced madarosis significantly impacts patient quality of life, yet current assessment methods rely heavily on subjective grading, limiting the evaluation of preventive strategies. This study presents a novel automated computer vision pipeline for objective longitudinal quantification of periocular hair density change and assesses its intra-session repeatability in a pilot clinical setting. The methodology integrates facial landmark detection, multi-temporal registration, and trimap deep learning-based hair segmentation with custom morphological filtering to isolate eyebrow hair structures from noise. The framework was applied to breast cancer patients undergoing anthracycline and taxane-based chemotherapy with localized cryotherapy. To evaluate the measurement repeatability, four standardized photographs (two with eyes open and two with eyes closed) were acquired and processed independently at each time point. Results confirmed the system’s sensitivity in tracking individual evolution, accurately capturing the contrasting density changes observed between subjects from baseline to follow-up. Although the longitudinal trajectories are presented as proof of concept of temporal tracking capability, the method exhibited relatively high precision, with intra-session standard deviations consistently remaining below 12% regardless of hair density, with slightly higher values observed at baseline in some cases. We conclude that this automated computer vision pipeline provides a robust, operator-independent metric for monitoring madarosis. By overcoming the limitations of manual grading, this tool represents a promising step toward establishing a reliable primary endpoint for future multicentre clinical trials once validated in larger cohorts.

## Introduction

Chemotherapy-induced alopecia (CIA) is one of the most common visible adverse effects of cancer treatment, affecting the physical traits and mental health of approximately two thirds of the patients receiving cytotoxic chemotherapy^1–4^. Pathophysiologically, chemotherapy damages rapidly dividing matrix keratinocytes in anagen hair follicles, leading to dystrophic anagen effluvium on the scalp and to varying degrees of eyebrow and eyelash loss^5–8^. Here, we present a novel automated computer-vision pipeline for objective quantification of periocular hair loss due to chemotherapy.

Hair loss can disturb the self-image and social identity of patients, and signals illness to others, leading to anxiety, depressive symptoms, and impaired quality of life^1–3,9^. Moreover, although most research has focused on scalp hair, madarosis (loss of eyebrows) induced by cancer treatment^6–8,10^ is of particular interest, since it affects ocular protection, facial expression and recognition, and perceived attractiveness^3,8^. Despite being a recurrent psychological concern for patients, madarosis is understudied^7^.

Over the last decade, scalp cooling has become the only preventive intervention for CIA cleared by the US Food and Drug Administration (FDA) and incorporated into international supportive care recommendations^4,11,12^. This regional cooling induces vasoconstriction, reduces local blood flow, and may decrease delivery of cytotoxic drugs to hair follicles, thereby mitigating follicular damage^13^.

Randomized and observational studies of automated scalp-cooling devices showed reductions in scalp alopecia for breast cancer patients receiving anthracycline and taxane-based chemotherapy^12–15^. However, since the eyebrows follicles lie outside the effective cooling field of these devices, clinical studies reported that madarosis remains common even among the patients that retained substantial scalp hair, highlighting that conventional cooling systems are not efficient in protecting facial hair^16^. Moreover, in contrast to scalp alopecia, eyebrow loss during anthracycline and taxane-based chemotherapy remains poorly studied and quantified despite its similar psychosocial relevance, where the facial expressions and the self-image of the patients are significantly altered. These features make chemotherapy-induced madarosis a distinctive supportive-care problems and justify the need for dedicated, eyebrow-specific quantitative endpoints.

To address this limitation, we implemented a localized eyebrow cooling protocol using cold gel eye masks in breast cancer patients receiving anthracycline-taxane regimens in a broader clinical setting registered at ClinicalTrials.gov (Identifier: NCT06955702; https://clinicaltrials.gov/study/NCT06955702) on 02-05-2025. Within this framework, we acquired standardized periocular images to assess the performance of the proposed computer vision pipeline.

Notably, both dermatologic and oncodermatologic reviews emphasize that there are no established, evidence-based guidelines for madarosis prevention in patients undergoing cancer treatment, to the best of the authors’ knowledge, and that available data are quite scarce and limited to case series^6–8^. Therefore, one of the major obstacles to advancing preventive strategies for chemotherapy-induced madarosis is the lack of objective methods for quantifying eyebrow hair loss and regrowth. The existing literature of CIA relies heavily on scalp-focused grading systems such as the Common Terminology Criteria for Adverse Events (CTCAE)^17^ or on patient self-report of hair loss as a binary or ordinal variable^9,18,19^. These methods cannot capture the regional differences, small density changes, or asymmetries relevant to eyebrows.

However, in recent years, the Brigham Eyebrow Tool for Alopecia (BETA) provides quantitative assessment of non-scalp hair change based on digital images, accounting for surface area and density^20^. They have demonstrated high inter- and intra-rater reliability in alopecia areata cohorts, but they have not yet been systematically validated in chemotherapy-induced periocular hair loss and appear to have seen limited application in this setting. In parallel, non-invasive imaging techniques such as trichoscopy and high-resolution digital photography have gained importance in the diagnosis and monitoring of hair and scalp disorders and can potentially be extended to eyebrows^21^.

Automated computer vision algorithms were used in a recent work in oculoplastic and ophthalmic surgery, and they could reliably segment the periocular region, detect landmarks, and quantify eyelid and brow morphology from standardized frontal photographs. For instance, computer vision systems were able to accurately measure palpebral fissure height, brow position, and eyelid area before and after upper eyelid surgery^22–24^. Moreover, AI-driven frameworks such as PeriorbitAI achieved high accuracy in automatically measuring eyelid and brow distances, proving the feasibility of camera-based assessment of periocular changes in real conditions^25^. Concurrently, digital image analysis has already been used to measure the results of several clinical trials of treatments for eyelash hypotrichosis, assessing chemotherapy-induced milphosis, where length and thickness variations are quantified from standardized photographs using a dedicated software^26,27^.

This shows that image-based quantification of eyebrow hair is both practical and sensitive to treatment effects. Nevertheless, to date, to the best of the authors’ knowledge, there is no validated computer vision pipeline specifically tailored to evaluate the preventive effects of localized cryotherapy, or to quantify eyebrow density changes induced by chemotherapy. Developing such a methodology is of paramount importance because it can provide an objective, reproducible, operator-independent metric that could potentially facilitate comparison across centres and trials in the future.

Furthermore, thanks to its detailed measurement, it can be a robust method for quantitatively measuring the periocular hair change over time (pre, during, and post-treatment). This leads to a deeper understanding of chemotherapy and localized cryotherapy effects and serves as a guide for optimizing cooling protocols to preserve periocular hair.

The aim of this work was to develop and technically assess a computer vision-based methodology for automatic periocular ROI detection, longitudinal eyebrow-density quantification, and repeatability analysis in breast cancer patients undergoing anthracycline- and taxane-based chemotherapy under a localized cryotherapy protocol. The cryotherapy setting provides the clinical context for image acquisition, whereas the primary objective of this study is methodological rather than therapeutic.

The remainder of this manuscript is structured as follows. Section  2 describes the study design, cryotherapy protocol, image acquisition, and the proposed computer vision methodology. Section  3 presents the experimental results and precision analysis of the automated pipeline, Sect.  4 discusses the implications, strengths, and limitations of the approach, and Sect.  5 concludes with perspectives for future clinical applications.

## Materials and methods

### Study design

This research reports a pilot methodological study focused on the technical assessment of an automated computer vision pipeline for longitudinal eyebrow-density quantification, performed on images acquired within a standardized clinical cryotherapy. While the patients followed a cooling protocol, the primary aim of this specific work is to demonstrate the technical feasibility and metrological reliability of the computer vision pipeline rather than the evaluation of treatment efficacy.

The cohort included two breast cancer patients scheduled to undergo anthracycline and taxane-based chemotherapy. The study was conducted within the Salamanca Health Area and Valdecilla University Hospital (Santander, Spain) to assess the methodology’s reproducibility across four clinically relevant time points: baseline prior to chemotherapy (T1), mid-treatment (T2), chemotherapy completion (T3), and 1-month follow-up (T4). These intervals were selected to capture the full trajectory of hair toxicity, monitoring the cumulative impact of the cytotoxic regimen from the initial healthy state through to the early post-treatment recovery phase).

### Cryotherapy mask

**Fig. 1:**
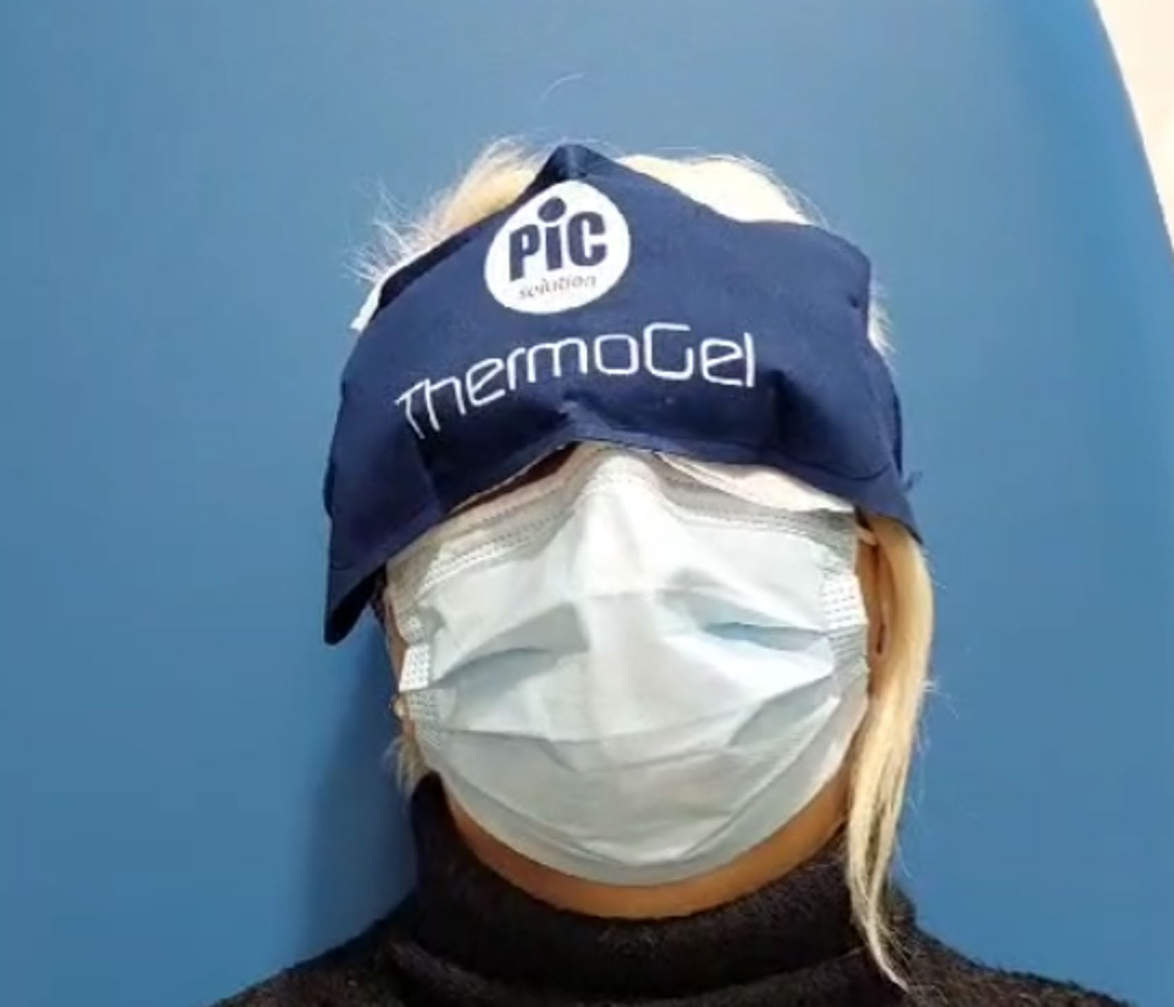
Placed cooling device during chemotherapy treatment.

The cryotherapy intervention utilizes a specialized gel-based cooling mask (Fig. 1). This device was selected based on a prior comparative study that evaluated various cooling systems to determine the most suitable option for maximizing anatomical coverage of the eyebrows while ensuring prolonged cold retention. To induce and maintain continuous vasoconstriction, the masks are pre-chilled to their freezing point before application. During the procedure, the masks are replaced immediately once the gel reverts to a liquid state to prevent thermal equilibration. The cooling protocol is initiated 15 min prior to the start of the anthracycline or taxane infusion and is maintained continuously throughout the administration, concluding 20 min post-infusion.

### Data acquisition and image processing

A digital camera (Canon EOS 700D) equipped with a 60 mm lens is used to take facial photographs of the participants; it is positioned approximately 1.5–2 m from the participants. The central focus point of the camera is placed between the eyebrows, and the participants are instructed to maintain a neutral facial expression and a steady head position without tilt or rotation to minimize blurriness. Images were acquired using flash in combination with maximized ambient light to enhance the visibility of fine hair structures (Table 1). Focus was manually verified through the device screen using digital zoom before the final capture. This acquisition protocol is designed to be easily reproducible in clinical practice; it does not require the use of a tripod or specialized photographic skills and could be adapted for use with consumer-grade devices, such as smartphones.

**Table 1:** Image acquisition parameters.

Parameter	Recommended range/value
Distance	1.5–2 m
Focal length	60 mm
Lighting	Maximum, ambient and diffused
Flash	Activated
Head Pose	Frontal, neutral gaze

For each participant, two images with eyes open and two with eyes closed (without squeezing eyelids) are taken at various phases of their treatment (T1-T4). The inclusion of both open and closed-eye configurations serves to introduce controlled anatomical variability in the periocular region, allowing for the assessment of the algorithm’s robustness against minor changes in facial expression. By assessing the consistency of the method across these distinct states, we evaluated whether eyebrow density quantification remains consistent regardless of the eyelid position. Furthermore, duplicate images for each state are acquired consecutively from the same position to ensure data redundancy and allow for the calculation of intra-session measurement precision.

### Method developed (Fig. 2)

**Fig. 2:**
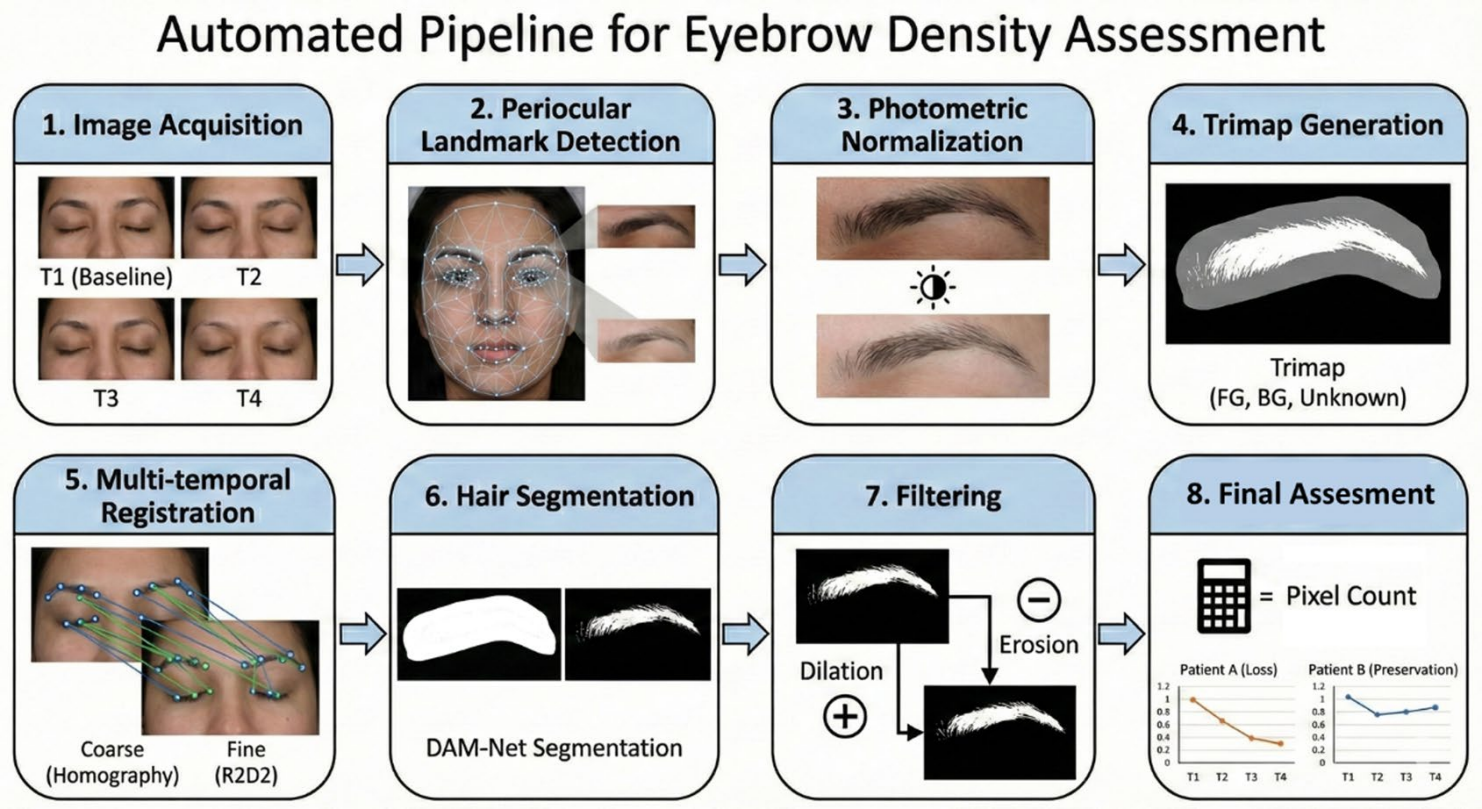
Overview of the automated computer vision pipeline for periocular hair quantification.

#### Periocular landmark detection

In order to obtain consistent images of the periocular regions of interest, MediaPipe Face Mesh^29^ is used to detect the periocular landmarks, using a resized representation of each image to stabilize inference, and the landmarks’ coordinates are mapped back to their original resolution.

**Fig. 3:**
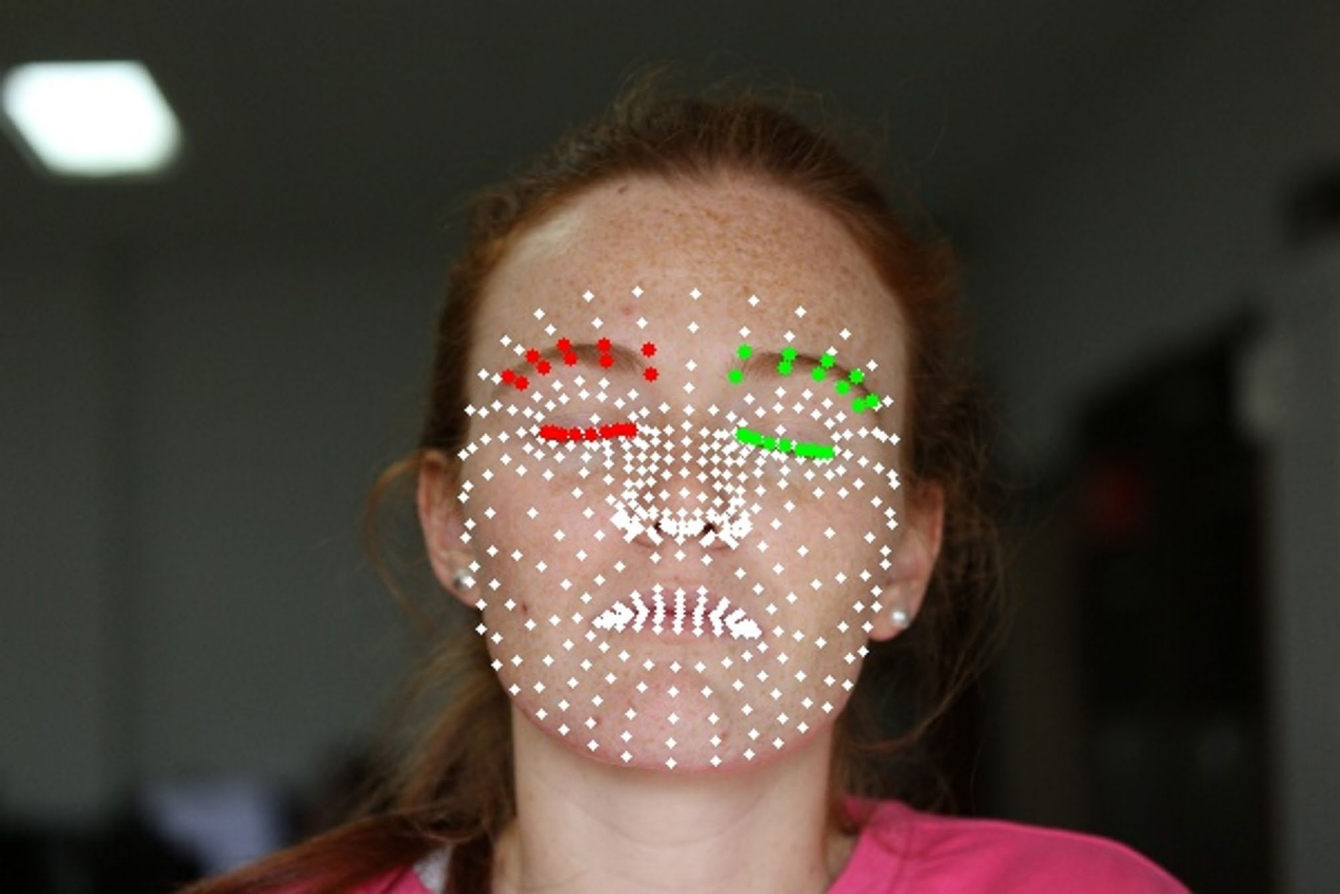
Facial landmarks detected by MediaPipe. Green and red points indicate the pre-defined MediaPipe indices used for ROI extraction, while white points represent unused landmarks from the general face mesh. The individual shown is a volunteer and not a study participant. Formal written consent for the publication of this image was obtained.

MediaPipe Face Mesh is a machine learning based facial landmark detector that can estimate a dense facial mesh (hundreds of landmarks), and we applied it in this study in static image mode (Fig. 3). For each image I of size W × H, a resized representation $\:{\mathrm{I}}^{\left(r\right)}$ with a fixed target width of 600 pixels is created. Although the computational load of high-resolution images is manageable for this dataset size, this rescaling is strictly necessary to stabilize landmarks inference, as the pre-trained model is optimized for lower-resolution inputs and may exhibit reduced accuracy or detection failures on full-resolution images.

To focus on the relevant anatomical areas and to stabilize the registration, a fixed subset of 38 landmarks (Fig. 3) was automatically extracted based on their topological IDs. This deterministic selection ensures that the periocular ROI is consistently localized across different subjects without any manual input.

The landmarks shown as “used” in Fig. 3 correspond to a predefined fixed subset of MediaPipe Face Mesh indices selected to delimit the periocular contour and eyebrow-support region for each eye. This subset is specified once in the implementation and applied automatically to all images. Hence, no manual landmark selection or adjustment is performed at the patient or timepoint level.

For each eye, the bounding region is computed from the landmark polygon, including the eyebrow structures. Hence, there are two sets of periocular landmarks indices that represent the contour around each eye e, defining a point cloud:

1 $$\:{P}_{e}^{\left(r\right)}={p}_{k}^{\left(r\right)}=\left({x}_{k}^{\left(r\right)},{y}_{k}^{\left(r\right)}\right);\:\:\:\:\:\:\:\:\:\:\:\:\:\:k\in\:{S}_{e}$$

Where $\:{S}_{e}$is the index set landmark associated with the corresponding eye $\:e\in\:\left\{\mathrm{1,2}\right\}$, and $\:\left({x}_{k}^{\left(r\right)},{y}_{k}^{\left(r\right)}\right)$ are the points coordinates of the resized images.

**Fig. 4:**
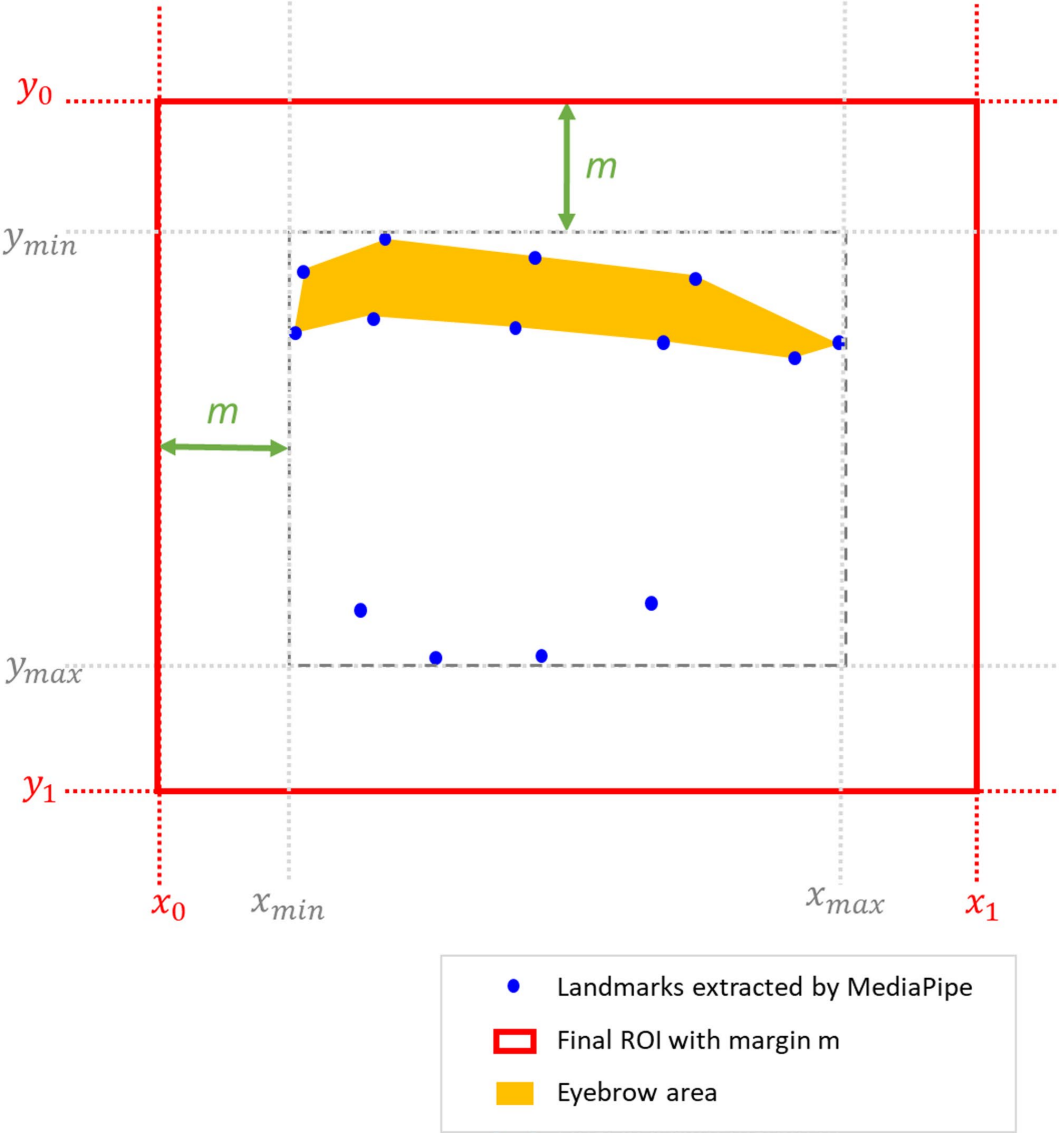
Schematic representation of the ROI extraction formulation. The blue points represent the detected periocular landmarks $\:{P}_{e}^{\left(r\right)}$ (Eq. 1). The dashed gray box indicates the tightest axis-aligned rectangle defined by the extrema $\:\left({x}_{min},{y}_{min}\right)$. The orange polygon shows the eyebrow area. The solid red box represents the final Region of Interest (ROI) after applying the expansion margin $\:m$, resulting in the coordinates $\:\left({x}_{0},{y}_{0}\right)$ used for cropping (Eqs. 4–5).

Then, the smallest axis aligned rectangle containing all the points in $\:{P}_{e}^{\left(r\right)}$ is computed to deduce the periocular region of interest, including a small margin $\:m\:$to account for the entire periocular hair region (Fig. 4).

Let us denote the regions of interest extrema coordinates for each eye as follow:

2 $$\:{x}_{min,e}^{\left(r\right)}=\underset{\left(x,y\right)\in\:{P}_{e}^{\left(r\right)}}{\mathrm{min}}x,\:\:\:\:\:\:{x}_{max,e}^{\left(r\right)}=\underset{\left(x,y\right)\in\:{P}_{e}^{\left(r\right)}}{\mathrm{max}}x$$

3 $$\:{y}_{min.e}^{\left(r\right)}=\underset{\left(x,y\right)\in\:{P}_{e}^{\left(r\right)}}{\mathrm{min}}y,\:\:\:\:\:\:\:\:{y}_{max,e}^{\left(r\right)}=\underset{\left(x,y\right)\in\:{P}_{e}^{\left(r\right)}}{\mathrm{max}}y$$

The real bounds including a fixed margin $\:m\:$in the resized coordinate system are given by:

4 $$\:{x}_{0,e}^{\left(r\right)}={x}_{min,e}^{\left(r\right)}-m,\:\:\:\:\:\:\:\:\:\:\:\:\:{x}_{1,e}^{\left(r\right)}={x}_{max,e}^{\left(r\right)}+m$$

5 $$\:\:{y}_{0,e}^{\left(r\right)}={y}_{min,e}^{\left(r\right)}-m,{\:\:\:\:\:\:\:\:\:\:\:\:\:y}_{1,e}^{\left(r\right)}={y}_{max,e}^{\left(r\right)}+m$$

At the end of this process, these bounds are mapped back to the original high-resolution image as $\:\left(x,y\right)=s\left({x}^{\left(r\right)},{y}^{\left(r\right)}\right)$, assuming a uniform scaling with a factor s$\:=W/{W}_{r}$, yielding:

6 $$\:{x}_{0,e}=s{x}_{0,e}^{\left(r\right)},{\:\:\:\:\:\:\:\:\:\:\:\:\:\:\:x}_{1,e}=s{x}_{1,e}^{\left(r\right)}$$

7 $$\:{y}_{0,\mathrm{e}}=s{y}_{0,\mathrm{e}}^{\left(r\right)},\:\:\:\:\:\:\:\:\:\:\:\:\:\:\:\:{y}_{1,\mathrm{e}}=s{y}_{1,\mathrm{e}}^{\left(r\right)}$$

The aspect ratio is preserved, so the same factor applies in $\:y\:$direction. The final high-resolution periocular ROI for eye $\:e\:$is then obtained by cropping the original image $\:I\:$as:

8 $$\:RO{I}_{e}=I\left({y}_{0,\mathrm{e}}:{y}_{1,\mathrm{e}};{\:x}_{0,\mathrm{e}}:{x}_{1,\mathrm{e}}\right)\:$$

In parallel, the landmarks coordinates were also rescaled to the original image coordinates using the same scaling method, $\:{p}_{k}=\left({x}_{k},{y}_{k}\right)=\mathrm{s}\left({x}_{k}^{\left(r\right)},{y}_{k}^{\left(r\right)}\right)$, and any point expressed in the full image coordinates system can be re-expressed in ROI local coordinates by $\:{p}_{k}^{\left(ROI\right)}={p}_{k}-in{i}_{e}$, where $\:in{i}_{e}=\left({x}_{0,\mathrm{e}},\:{y}_{0,\mathrm{e}}\right)$.

It is worth noting that a combined periocular ROI may be computed using the union of both eye landmark sets with the same margin expansion:

9 $$\:{P}_{comb}^{\left(r\right)}={P}_{1}^{\left(r\right)}\cup\:{P}_{2}^{\left(r\right)}$$

#### Photometric normalization

The luminance within the periocular regions of interest is normalized in the HSV colour space, where for each ROI the image is converted from the OpenCV BGR representation to HSV and decomposed into Hue (H), Saturation (S), and Value (V) channels. We calculate the mean intensity $\:{{\upmu\:}}_{t}$ of the current timepoint and compare it against a reference luminance $\:{V}_{ref}$, defined as the mean intensity of the baseline image $\:\left(T1\right)$. A global offset is then applied to align the brightness of the current image with the baseline. Finally, a numerical saturation step is applied to restrict the resulting pixel values to the valid 8-bit range [0, 255], preventing dynamic range overflow (white saturation) or underflow (black crushing) artifacts. Hence, we kept H and S invariant while replacing V with $\:{V}_{norm}$ and converted back to BGR to photometric normalization of the regions of interest.

The luminance normalization in the HSV color space^28^ follows established conventions in digital dermatology and skin lesion analysis^29,30^. By decoupling the luminance (V channel) from the chromatic information (H and S channels), we attenuate shading artifacts and inconsistent illumination that often degrade automated hair segmentation. This deterministic preprocessing step ensures that longitudinal changes in pixel count reflect true biological hair density variations rather than transient fluctuations in ambient lighting conditions.

#### Trimaps generation

**Fig. 5:**
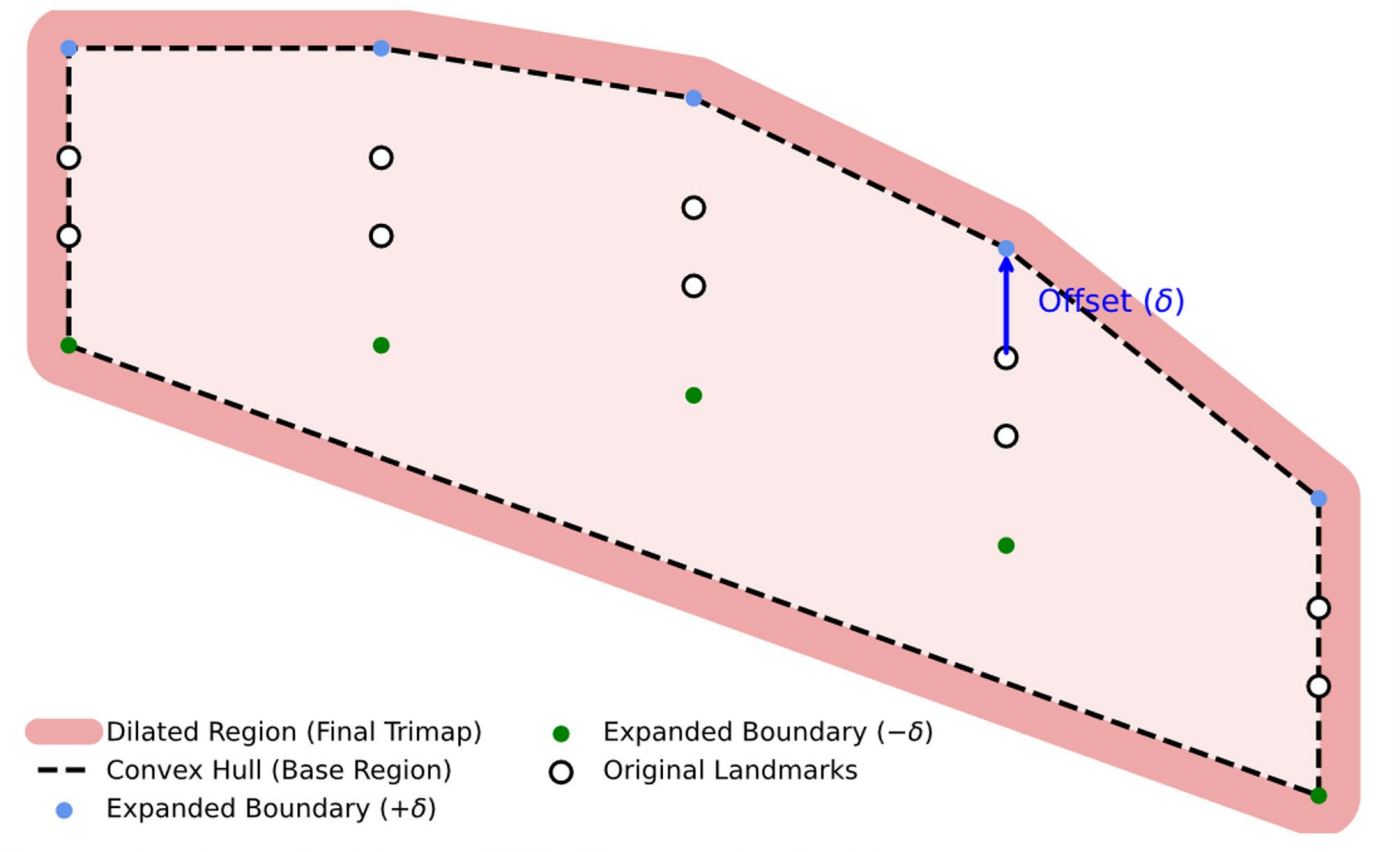
Automated trimap generation pipeline. The process begins with the original periocular landmarks (white points), which are vertically expanded by an adaptive offset $\:\pm\:{\updelta\:}$ (blue/green points). A Convex Hull (dashed line) is computed to enclose the region, followed by a morphological dilation (red area) to define the final “unknown” region for the segmentation network.

To segment the structure of eyebrow hairs, we employ the the Deep Aggregation Matting Network (DAM-Net)^31^ architecture, which relies on a trimap as input. A trimap is a tripartite mask that partitions the image into three distinct zones: definite foreground (white), definite background (black), and an unknown transition region (grey). This map serves as a crucial prior, directing the network to focus its boundary refinement efforts exclusively on the unknown region where the complex hair-skin boundaries are located. We developed an automated pipeline to generate these trimaps based on the geometric properties of the subject’s face:

1. Adaptive Vertical Expansion: First, we extract the superior and inferior periocular landmarks defining the eyebrow arch. To ensure the region of interest captures the full width of the eyebrow—including potential stray hairs—we apply an adaptive vertical offset ($\:{\updelta\:}$). The upper landmarks are shifted upward ($\:+{\updelta\:}$) and the lower landmarks downward ($\:-{\updelta\:}$). This offset is proportional to the subject’s facial dimensions, ensuring anatomical consistency across different patients. The offset ($\:{\updelta\:}$) is calculated as 30% the eyebrow area height (calculated using MediaPipe landmarks, as shown in Fig. 4).
2. Convex Hull Construction: From this expanded set of points, we compute the Convex Hull, which generates the tightest polygonal boundary enclosing all shifted landmarks. This step creates a coherent, continuous region that robustly covers the eyebrow structure.
3. Morphological Dilation: Finally, to account for any remaining irregularities or hairs extending beyond the estimated hull, we apply a morphological dilation to the polygon. This creates a final “safety margin” (dilated region), ensuring that all relevant pixels are included within the unknown region of the trimap for accurate processing (Fig. 5). This generation is performed on the baseline (T1) images and propagated to subsequent timepoints via spatial registration, providing a standardized region of interest unaffected by hair loss progression.

This baseline-referenced strategy was chosen to preserve anatomical consistency across timepoints, even when visible eyebrow density decreases substantially. Its main advantage is that the same periocular support region is evaluated longitudinally, reducing bias from time-varying manual or adaptive ROI definition. However, when eyebrow boundaries change significantly, the propagated trimap may reflect baseline anatomical assumptions more strongly than the visible hair contour at later stages. To minimize this risk, the expanded landmark set, convex hull, and final dilation step were deliberately designed to provide a conservative safety margin around the eyebrow region, and all outputs were visually reviewed during the pilot analysis to identify potential registration or segmentation failures.

#### Two stage multitemporal registration

The use of hierarchical coarse to fine registration methodologies is well established in the broad image registration literature, where an initial global alignment is refined by a second one that is designed to correct local residual mismatches. This strategy is adopted here because stable comparison across time requires both anatomically meaningful initialization and thorough subsequent corrections.

The ROIs images are aligned to the baseline T1 coordinate system through two consecutive projective mapping (homographies); the first one is a coarse alignment estimated from the corresponding periocular landmarks, while the second uses the Repeatable and Reliable Detector and Descriptor (R2D2) keypoints^32^ learned features matched to the reference baseline to correct any residual misalignment. The second registration stage is then introduced because the first projective mapping corrects only the global geometric discrepancies but may leave residual local misalignments due to eyelid tension or any subtle expression related changes that are addressed by the second homography. All the final registered regions of interest are then computed in the baseline coordinates system.

The adoption of a coarse-to-fine registration strategy is a well-documented paradigm in computer vision to manage significant spatial displacements while maintaining sub-pixel accuracy. Traditional approaches have long utilized global geometric constraints followed by local feature refinement^33–35^.

Let $\:{I}_{e}^{\left(t\right)}$ be the cropped ROI at time $\:t\:$for eye $\:e$, and $\:{I}_{e}^{\left(T1\right)}$ the corresponding baseline ROI. As mentioned above, MediaPipe provides $\:k\:$periocular landmarks in full image coordinates $\:{\mathrm{p}}_{k,e}^{\left(t\right)}$ and $\:{\mathrm{p}}_{k,e}^{\left(T1\right)}$. However, since the registration is done in the local ROI coordinates, the landmarks are converted to this local coordinate system ($\:{p}_{k}^{(ROI,\:t)}$ and $\:{p}_{k}^{(ROI,T1)}$) by simply subtracting the ROI top left origin $\:{ini}_{e}^{\left(t\right)}$ and $\:{ini}_{e}^{\left(T1\right)}$, respectively.

Therefore, the first coarse homography $\:{H}_{1,e}^{\left(t\to\:T1\right)}\:\in\:\:R\left(3\times\:3\right)$, is estimated from these landmarks such that:

10 $$\:{p}_{k,e}^{\left(ROI,T1\right)}\sim\:{H}_{1,e}^{\left(t\to\:T1\right)}{p}_{k,e}^{\left(ROI,t\right)}$$

This results in a coarse projective warp of the follow-up ROI into the baseline grid:

11 $$\:{I}_{e}^{\left(t,tr1\right)}=Warp\:\left({I}_{e}^{\left(t\right)},{H}_{1,e}^{\left(t\to\:T1\right)}\right)$$

Where tr1 refers to the transformed ROI after this initial landmark-based warp into the T1 frame. $\:Warp\left(.\right)$ is implemented using a perspective warp. For consistency, the same transform is applied to the associated eyebrow trimaps.

Since this landmark-based homography leaves residual misalignments due to local skin deformations, eyelid tension, etc. the second homography using the learned focal features is needed. R2D2 is run on both the baseline and the coarsely aligned ROIs producing N matched keypoints $\:{q}_{i,e}^{\left(T1\right)}\leftrightarrow\:{q}_{i,e}^{\left(tr1,t\right)}$. Hence, a second homography $\:{H}_{2,e}^{\left(tr1\to\:T1\right)}$ can be estimated:

12 $$\:{q}_{i,e}^{\left(T1\right)}\:\sim\:\:{H}_{2,e}^{\left(tr1\to\:T1\right)}{q}_{i,e}^{\left(tr1,t\right)};\:\:\:\:\:\:\:\:\:\:\:\:\:\:\:\:i=1,\dots\:,N$$

It is then applied to obtain the final refined registered ROI:

13 $$\:{I}_{e}^{\left(t,tr2\right)}=Warp\left({I}_{e}^{\left(t,tr1\right)},\:{H}_{2,e}^{\left(tr1\to\:T1\right)}\right)$$

The corrected trimaps are obtained similarly. Therefore, the total mapping from the follow-up ROI coordinates to the baseline coordinates system is given by the matrix product:

14 $$\:\:H_{{total,e}}^{{\left( {t \to \:T1} \right)}} = \:H_{{2,e}}^{{\left( {tr1 \to \:T1} \right)}} \cdot \:H_{{1,e}}^{{\left( {t \to \:T1} \right)}}$$

This shared baseline coordinate system is necessary for segmentation outputs comparability across time.

The registration accuracy was quantitatively assessed using the Root Mean Square Error (RMSE) across the two stages of the pipeline.

#### Trimap guided hair segmentation

To accurately capture the fine-grained structure of periocular hair, which often contains semi-transparent strands and complex boundaries, we formulate the task as an image matting problem rather than simple binary segmentation. We employ DAM-Net, a deep learning architecture specifically designed to estimate the opacity (alpha-matte) of foreground objects within the ‘unknown’ region of the trimap.

The DAM-Net architecture follows an encoder-decoder structure but introduces a distinctive Deep Aggregation Module (DAM). This module effectively fuses high-level semantic features (encoding the global shape of the eyebrow) with low-level texture features (encoding individual hair strands). By taking the periocular ROI and the previously generated trimap as inputs, the network focuses its computational attention solely on the transition regions defined by the trimap. The model outputs a continuous per-pixel probability map, where each pixel value $\:p\in\:\left[\mathrm{0,1}\right]$ represents the likelihood of belonging to the hair structure. This soft-segmentation approach allows for the preservation of fine hair details that would typically be lost in binary thresholding methods.

In this study, DAM-Net was utilized as a pre-trained model, as the creation of a manual pixel-level ground truth for fine hair structures was deemed unfeasible due to high density and sub-pixel dimensions. A primary objective of this study was to evaluate the suitability of this general-purpose matting architecture for the specific domain of periocular hair segmentation.

#### Post-processing and morphological filtering

Following the semantic segmentation via DAM-Net, a custom automated filtering pipeline was implemented to isolate the hair structure and eliminate false-positive background artifacts. The raw probability maps were first converted to grayscale and binarized using a high-sensitivity low-intensity threshold ($\:\tau\:=1$) to ensure the preservation of maximum feature detail, including fine hair strands. A sensitivity analysis demonstrated the robustness of this parameter: increasing the threshold tenfold ($\:\tau\:=10$) resulted in a 7.2% variation in total hair pixel detection, indicating high algorithmic stability.

Hence, each binarized output (B) is defined as follows:

15 $$\:B(x,y)=\left\{\begin{array}{cc}1,&\:\mathrm{if\:}{M}_{prob}(x,y)>\tau\:\\\:0,&\:\mathrm{otherwise}\end{array}\right.$$

To refine the morphological structure of the mask, a two-stage operation was applied. First, a morphological opening was employed to suppress high-frequency “salt” noise using an elliptical kernel with dimensions proportional to the eyebrow area (as shown in Fig. 4, given by MediaPipe landmarks) size (5% of the eyebrow area height). This was immediately followed by a dilation operation to consolidate fragmented regions and ensure the structural connectivity of the primary hair component. The dilation utilized an elliptical kernel of 7% of the eyebrow height. These proportional settings ensure that the measurement sensitivity remains uniform across all clinical time points.

Subsequently, a Connected Component Analysis (CCA) with 8-connectivity was performed to label the set of discrete objects $\:\mathcal{C}=\{{C}_{1},{C}_{2},\dots\:,{C}_{N}\}$ within the binary mask. The algorithm automatically identified the primary hair component ($\:{C}_{main}$) as the one with the maximal area:

16 $$\:{C}_{main}={arg}\underset{{C}_{i}\in\:\mathcal{C}}{{max}}\left(\mathrm{Area}\left({C}_{i}\right)\right)$$

To account for disjointed but relevant hair patches, secondary components were retained only if they satisfied a dual spatiotemporal criterion based on their size and proximity to the main component’s centroid ($\:{{\upmu\:}}_{main}$). Formally, the final optimized mask ($\:{M}_{opt}$) is defined as the union of all components $\:{C}_{i}$ that satisfy the retention function $\:\mathcal{R}\left({C}_{i}\right)$:

17 $$\:{M}_{opt}={\bigcup\:}_{i=1}^{N}\{{C}_{i}\mid\:\mathcal{R}\left({C}_{i}\right)\mathrm{\:is\:true}\}$$

$$\:\mathcal{R}\left({C}_{i}\right)\:=\left\{\:\begin{array}{c}\:True\:if\:{C}_{i}={C}_{main}\:\\\:True\:if\:\left|{\mu\:}_{i}\:-\:\:{\mu\:}_{main}\right|\:\\\:False\:otherwise\end{array}\right.\:<\:{\tau\:}_{dist}\:\wedge\:\:\mathrm{A}\mathrm{r}\mathrm{e}\mathrm{a}\left({C}_{i}\right)>{\tau\:}_{area}\:\:\:\:\:\:\:\:\:\:\:\:\:\:\:\:\:\:\:\:\:\:\:\:\:\:\:\:\:\:\:\:\:\:\:\:\:\:\:\:$$.

Where$\:\:{{\upmu\:}}_{i}\:$denotes the centroid of the component $\:{C}_{i},\:\:\left|{\mu\:}_{i}\:-\:\:{\mu\:}_{main}\right|\:$represents the Euclidean distance between the two centroids, the thresholds ($\:{\tau\:}_{dist}\:$and $\:{\tau\:}_{area})\:$formulated as adaptive ratios relative to the eyebrow dimensions. Specifically, $\:{\tau\:}_{dist}\:$is set to equal the 50% eyebrow height, with $\:{\tau\:}_{area}$ is defined as 1% of the area of a bounding square with sides equal to the eyebrow height. Components failing to meet these criteria were discarded as background noise. This scale-invariant approach ensures that the selection of relevant hair structures is guided by the subject’s unique anatomy rather than fixed pixel counts, effectively mitigating the risk of data-driven overfitting. Furthermore, utilizing the vertical height of the eyebrow arch as a reference dimension has demonstrated greater robustness than its horizontal length as it is less affected by perspective distortions or inter-subject variability.

It is worth noting that while these thresholds may be further refined in future developments, the primary objective of the proposed methodology is to evaluate the longitudinal trajectory of hair density. By focusing on relative changes rather than absolute counts, potential systematic errors are consistently applied across all time points, thereby minimizing their impact on the observed percentage evolution.

#### Final hair quantification

For each patient, timepoint and eye, the segmentation masks are binarized using a fixed threshold to distinguish between background and foreground pixels. The number of foreground pixels, representative of the eyebrows, is given by:

18 $$\:A=\sum\:_{x,y}B(x,y)$$

It is a consistent metric since all the regions of interests are registered to the same baseline (T1) coordinate frame, providing a comparable longitudinal measure across all sessions (T1-T4). Eyebrow’s hair loss during chemotherapy can then be expressed as a percent change (%) relative to the baseline (T1) as:

19 $$\:{\Delta\:}\left(t\right)=100\left(1-\frac{A\left(t\right)}{A\left(T1\right)}\right)\:$$

Here, the eyebrow hair density is defined as the proportion of foreground pixels assigned to eyebrow hair within a standardized and temporally registered periocular ROI. These metric captures then a 2D projected hair coverage in the image plane and not the follicular density itself, or volumetric hair mass. Although this standardized acquisition procedure reduces variability, $\:{\Delta\:}\left(t\right)$ may still be influenced by factors such residual cosmetic products and grooming. Therefore, we ensured that the selected patients had no such esthetical changes during the entire trial time (T1-T4). However, it is worth noting that the longitudinal percentage changes $\:{\Delta\:}\left(t\right)$ should be interpreted as image-based changes in visible hair coverage rather than a strict biological count of follicles or hairs.

### Intra-session repeatability assessment

To assess the precision of the automated pipeline, four images were captured per timepoint (two with closed eyes and two with open eyes). The four sets of images were processed independently. Since these captures were obtained within the same imaging session and before any meaningful biological change could occur, this analysis evaluates intra-session measurement repeatability independently of any potential intervention effect. By contrast, longitudinal changes across T1–T4 are presented descriptively to illustrate temporal tracking capability rather than to infer cryotherapy efficacy. The resulting hair density percentages were compared to calculate the intra-session standard deviation (Std). A low standard deviation across these four consecutive captures indicates high repeatability of the measurement method, isolating the algorithm’s performance from transient noise or capture artifacts.

To evaluate the metrological reliability of the automated pipeline, the Intraclass Correlation Coefficient (ICC) was employed to assess the technical stability of the density metrics across repeated captures within the same imaging session. Additionally, the Standard Error of Measurement (SEM) was calculated to quantify the technical precision of the system, identifying the degree of variation attributable to capture or algorithmic noise rather than biological change. Finally, the Minimum Detectable Change (MDC) was determined to establish a statistical threshold, defining the minimum percentage change required to distinguish true biological hair loss from inherent measurement variability in a clinical setting.

This work focuses primarily on intra-session repeatability and metrological reliability of the final density metric. Thus, it does not constitute a full segmentation accuracy study against pixel-level manual annotations. However, to assess the accuracy of the results, the resulting metrics of this computer-vision pipeline were compared to an expert BETA scoring that was additionally performed on the images.

## Implementation details

The algorithm underlying the computer vision pipeline described above is open access and available in TIDOP’s GitHub repository: https://github.com/TIDOP-USAL/crioma_madarosis-quantification.

The automated pipeline was implemented in Python 3.12 using the PyTorch framework. For eyebrow segmentation, the DAM-Net model was used with its original architecture weights, while the R2D2 descriptor was implemented using the pre-trained “r2d2_WASF_N16.pt” model. The pipeline was executed on a system running Windows 11 equipped with an Intel Core i9-10900X CPU, 32 GB of RAM, and an NVIDIA RTX A2000 GPU. Under this configuration, the total computational runtime for a complete patient longitudinal dataset (comprising 16 high-resolution images across four timepoints) is approximately 3.5 min, demonstrating the system’s feasibility for clinical integration.

## Experimental results

**Fig. 6:**
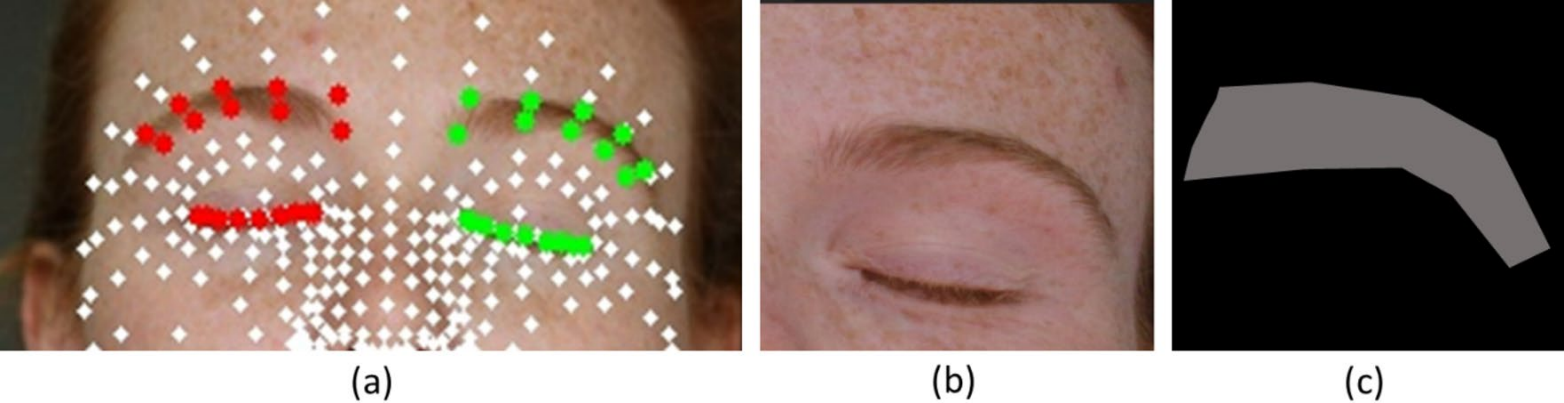
Obtainment of periocular landmarks (**a**), generated ROI image (example for left eye only) (**b**) and generated trimap (**c**). The individual shown is a volunteer and not a study participant. Formal written consent for the publication of this image was obtained.

Following the methodology detailed above, the analysis was conducted on four datasets obtained from two patients. For each subject, the pipeline successfully registered and segmented the periocular regions across the full treatment timeline (Fig. 6).

### Registration

**Fig. 7:**
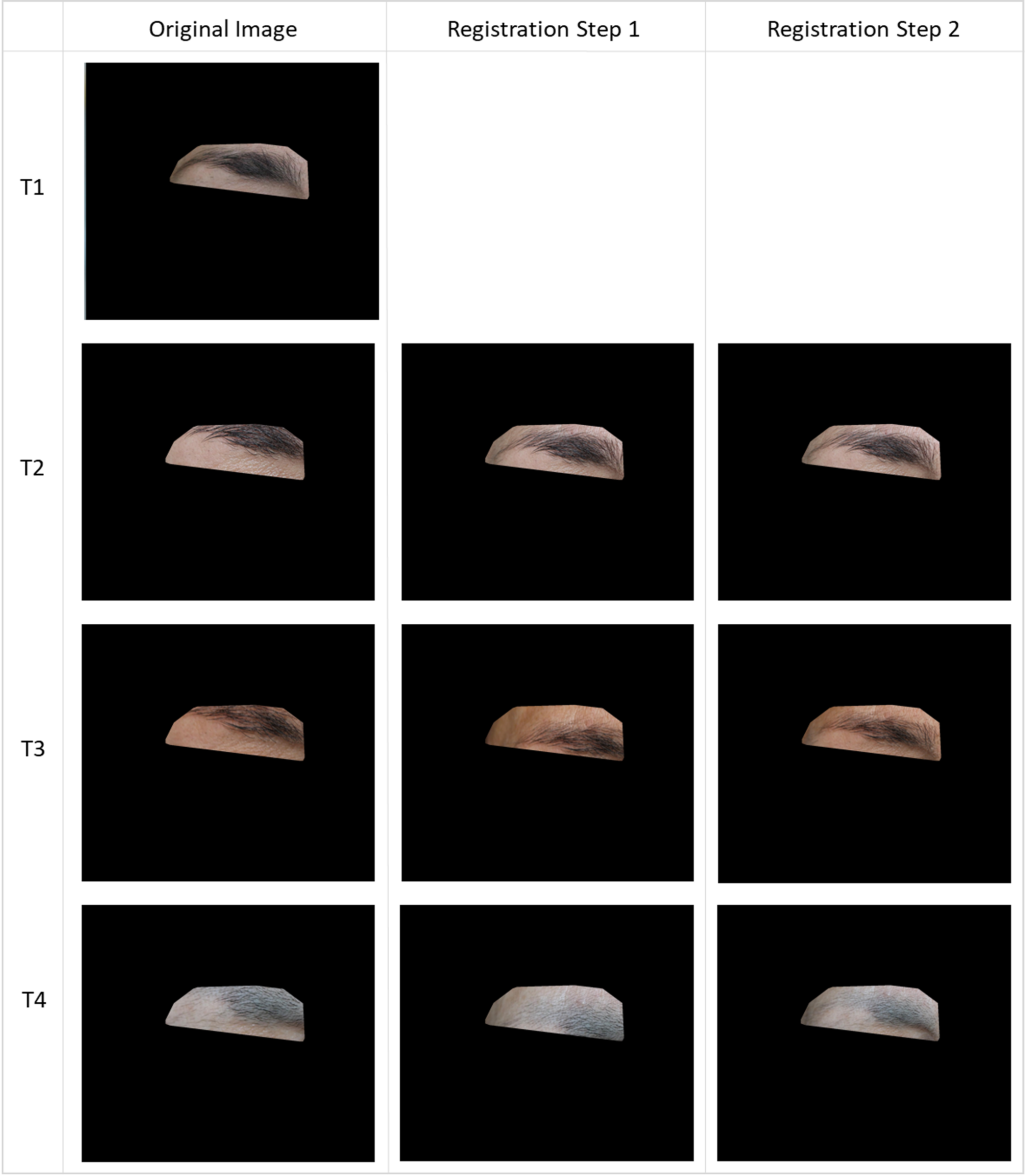
Registration of images for patient 2, visible areas correspond to trimaps. Only eyebrow areas are shown to preserve anonymity of the patient.

The proposed coarse-to-fine registration scheme demonstrated high precision in aligning longitudinal periocular images. The initial landmark-based homography provided a stable global alignment yet retained a mean RMSE of 6.62px. The subsequent fine-registration stage effectively corrected local deformations, reducing the mean RMSE to 1.88 px (Table 2). This sub-pixel level of precision is critical for the accurate quantification of hair density, as it ensures that the temporal comparison is performed on the exact same anatomical region. Registration results for patient 2 can be seen in Fig. 7.

**Table 2:** Quantitative assessment of the two-stage registration pipeline.

Registration stage	Mean RMSE (px)	Max RMSE (px)
Step 1 (Coarse)	6.62	11.99
Step 2 (Fine)	1.88	2.11

### Hair loss measurement

The analysis of the collected data demonstrates a high degree of consistency of the method across all time points (T1–T4). As shown in Table 3, the variability of the measurements remained low throughout the study. The standard deviation (Std) for patient 1, is below 9% for all time points, with a maximum of 8.64%. For patient 2 Std peaked at 10.61% at baseline (T1) and subsequently remained largely below 10 for the remaining time points. These results indicate a robust measurement method with low dispersion.

The analysis of individual eyes further supported the intra-session repeatability of the measurements. Although a maximum standard deviation of 11.12% was recorded for patient 2 at T1, this value remains within acceptable limits. Furthermore, the standard deviations for individual eyes were consistently comparable to the combined values, indicating a high degree of symmetry and reliability between both eyes.

Regarding the longitudinal density values, distinct temporal trajectories were observed between the two participants. While both patients started at a normalized baseline of 100.00%, Patient 2 maintained higher relative density values over time (86.65% at T2 and ending with 59.51% at T4) than Patient 1. These trajectories are presented here as proof-of-concept examples showing that the pipeline can capture heterogeneous longitudinal patterns under real clinical conditions. Given the pilot sample size (*N* = 2), no inference regarding cryotherapy efficacy can be drawn.

**Table 3:** Evaluation of measurement precision: mean hair density (%) for individual and combined eyes, and Intra-session standard deviations (%), calculated using Eq. 19.

	T1	T2	T3	T4
Patient 1				
Percentage	100.00	66.67	69.02	9.40
Std	6.39	7.64	8.64	4.04
Std eye 1	8.29	7.93	8.38	1.93
Std eye 2	3.99	2.76	8.99	4.45
Patient 2				
Percentage	100.00	86.65	83.53	59.51
Std	10.61	4.38	4.87	5.30
Std eye 1	11.12	3.66	5.95	2.48
Std eye 2	9.78	2.43	4.44	7.67

### Comparison with BETA

**Fig. 8:**
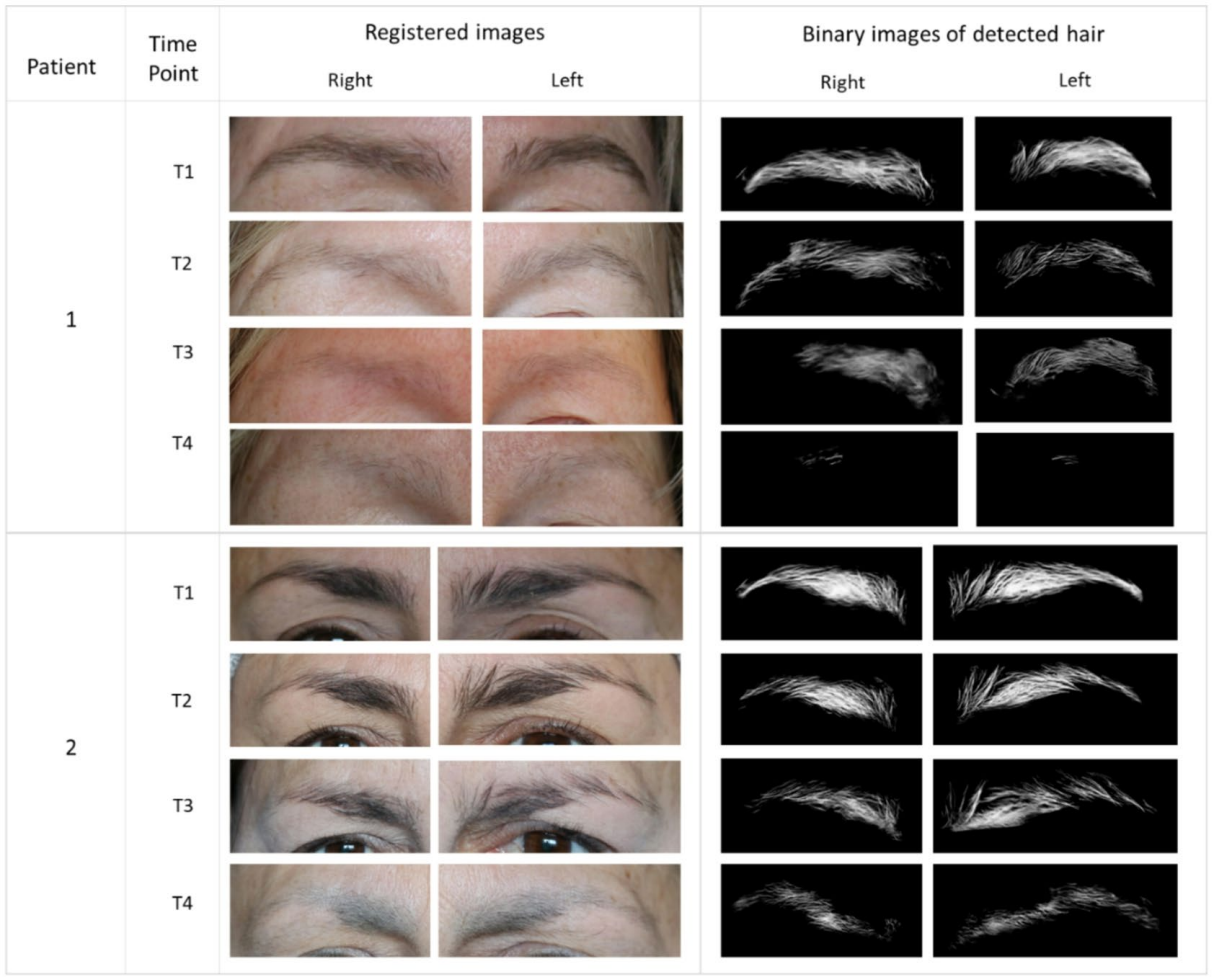
Registered images and binary images of detected hair for both patients. Images are cropped to preserve the anonymity of the patients.

The comparison with BETA was conducted to assess the preliminary concordance of the automated measurement system with an expert clinical scoring system. A clinical expert computed the BETA scores for all acquired images (Fig. 8) to serve as the clinical reference for comparison standard and the resulting data are summarized in Table 4.

**Table 4:** Comparison with BETA. Percentages of BETA with relation to T1.

	T1	T2	T3	T4
Patient 1				
Our method	100	66.7	69.0	9.4
BETA				
Score	6	3.8	1.9	1.5
Percentage	100	63.3	31.7	25.0
Patient 2				
Our method	100	86.7	83.5	59.5
BETA				
Score	6	5.55	5.1	3.4
Percentage	100	92.5	85.0	56.6

The analysis of Table 4 reveals a high correlation for patient 2 but a significant discrepancy between the two methodologies regarding patient 1 at the T3 and T4. While Fig. 8 shows that there is no substantial visual change between T2 and T3, the BETA score suggests a major decline. This drop occurs because the expert assigned a density score of 1, while previous images received and score of 2. This transition stems from the discrete nature of the ordinal scale, where a single-point change results in a substantial percentage shift that may not align with continuous changes in hair density. Such variations illustrate the inherent differences between categorical grading and continuous pixel-based quantification. By providing a continuous metric, the automated pipeline offers increased sensitivity to subtle density variations that may be masked by the coarser increments of traditional scoring systems.

To evaluate the metrological reliability of the automated pipeline, the Intraclass Correlation Coefficient ($\:ICC$) was calculated using a two-way random-effects, absolute agreement, single-rater model ($\:IC{C}_{\mathrm{2,1}}$). Additionally, the Standard Error of Measurement (SEM) was determined as $\:Std\times\:\sqrt{1-ICC}$ to quantify technical precision, and the Minimum Detectable Change (MDC) was defined as $\:SEM\times\:1.96\times\:\sqrt{2}$ to establish the statistical threshold required to distinguish true biological hair loss from measurement noise.

The pooled analysis for eyebrow density percentage yielded an ICC of 0.971 (95% CI: 0.941–0.988), indicating excellent reliability. In comparison, while BETA reports a high total ICC (~ 0.90), its performance is significantly lower when disaggregated: it achieves an ICC of 0.83 for density and only 0.58 for surface area. Crucially, BETA’s density metric relies on a restricted ordinal scale (0–3), which inherently “masks” or smooths measurement errors by forcing observations into a few discrete categories. Our pipeline overcomes this limitation by providing a continuous percentage metric, which offers superior granularity and sensitivity. Despite the much higher resolution of our scale—which typically makes high ICC values harder to achieve—the system maintains a SEM of 4.81% and an MDC of 13.24%. These results confirm that the framework is not only more reliable than current manual standards but also robust enough for longitudinal clinical monitoring where subtle density changes are paramount.

## Discussion

The primary objective of this study was to develop and technically evaluate an automated, objective methodology for quantifying eyebrow density changes in cancer patients undergoing chemotherapy in a pilot, proof-of-concept setting. While the existing literature relies heavily on subjective grading systems or patient self-reports^9,18,19^, we present a robust computer vision pipeline capable of providing precise, continuous metrics. The results obtained from the pilot application of this tool demonstrate its high internal validity and its potential to serve as a standard metric for future large scale clinical trials assessing preventative interventions, such as localized cryotherapy.

Our findings indicate that the proposed pipeline, combining landmark detection with deep learning-based segmentation, offers a high degree of stability in the measurement of facial hair.

As evidenced in Table 3, the measurement variability remained consistently low throughout the study. The standard deviation (Std) for patient 1 was below 9% across all time points. Similarly, while patient 2 showed a maximum standard deviation of 10.61% at baseline, subsequent measurements remained largely below 10%. This low dispersion suggests that the algorithm is robust against image noise and minor variations in head pose. Furthermore, the standard deviations for individual eyes were consistently comparable to the combined values.

An effective measurement tool must be sensitive enough to detect substantial hair loss. The distinct trajectories of the two participants in this study served as an ideal stress test for our methodology. The system accurately tracked the progression of patient 1, quantifying a steep decline to 9.40% at the follow-up stage. Conversely, it successfully quantified the preservation in patient 2, who retained 59.51% of hair density at the same stage.

The ability of the pipeline to distinguish these contrasting longitudinal trajectories with high precision confirms its utility for monitoring periocular hair change over time. Unlike binary or ordinal scales (e.g., CTCAE) which use coarse discrete categories to classify patients’ alopecia severity degree^17^, our method provides a percentage of loss. This level of detail is essential for future research, as it allows for the precise calculation of effect sizes in pilot studies. However, it is worth noting that in this pilot setting, such trajectories should not be interpreted as evidence of treatment effect.

While tools such as BETA provide quantitative assessments of eyebrow involvement in alopecia areata, they have not yet been systematically validated in chemotherapy-induced periocular hair loss and appear to have seen limited application in this setting. Furthermore, manual or semi-automated methods used in previous trials are labour-intensive and prone to inter-rater variability. Crucially, our automated pipeline offers superior measurement granularity compared to BETA. Whereas BETA relies on a limited ordinal scale (0–6) to estimate density, our method quantifies hair presence as a continuous percentage. This allows for greater sensitivity to subtle changes, with a demonstrated precision reflected by a standard deviation consistently around or below 10%. By automating the segmentation and implementing a custom filtering algorithm based on spatial and area thresholds, our approach eliminates operator dependency. This reproducibility would be critical for prospective multicenter trials where standardization across different clinical settings will be required.

## Limitations and perspectives

Several limitations must be addressed to contextualize the findings of this technical pilot study. First, while the small sample size of two patients (*N* = 2) provided sufficient data to establish excellent technical reliability, it remains insufficient to draw definitive conclusions regarding the clinical efficacy of the cryotherapy mask or the broader clinical reach of the results. To maintain high segmentation accuracy, individuals with eyebrow makeup or micro-pigmentation were strictly excluded, meaning the generalizability of the pipeline across a wider range of ethnicities, skin tones, and hair colours—particularly lighter shades—remains to be investigated in larger and more diverse cohorts. Furthermore, the current analysis successfully establishes the intra-session repeatability of the framework within a single clinical encounter, which effectively separates technical variability from biological change. These results provide a valuable proof-of-concept by demonstrating the technical feasibility and metrological reliability of the pipeline within this pilot setting, establishing a solid foundation for future clinical endpoint readiness. While the current scope focuses on this initial proof-of-concept and does not yet address inter-session factors such as changes in lighting, different days, or various operators, it confirms the framework’s potential for robust longitudinal monitoring.

Looking forward, while this work prioritized the technical assessment of eyebrow density repeatability, future research will extend this methodology to include systematic eyelash quantification (milphosis) to provide a more comprehensive periocular clinical assessment. The successful pilot assessment of this tool provides a methodological foundation to explore the use of this automated computer vision pipeline in larger randomized cohorts. By using this objective metric as a primary endpoint, future studies will rigorously evaluate whether localized cryotherapy can significantly reduce chemotherapy-induced madarosis and will correlate these objective density metrics with patient-reported outcomes on quality of life.

Future research will prioritize evaluating inter-session reproducibility to account for day-to-day variations in lighting and different operators, as well as assessing cross-device performance to ensure the algorithm’s robustness across various camera systems and mobile health platforms. Furthermore, conducting multicenter studies with larger and more diverse cohorts will be essential to confirm the framework’s clinical utility as a standardized, objective endpoint for monitoring chemotherapy-induced madarosis in broader oncology trials.

This research is a proof-of-concept study focused on the technical repeatability of a computer vision pipeline. While the broader trial was registered at ClinicalTrials.gov on May 2, 2025 (NCT06955702), the specific datasets used for this technical analysis were acquired in February 2025 under active ethical oversight. The registration timing was subject to administrative cycles related to the project’s funding (Plan TCUE 2024–2027).

## Conclusion

This pilot study presents a computer vision framework designed for objective longitudinal quantification of chemotherapy-induced periocular hair loss, and shows that the resulting metric has promising intra-session repeatability under standardized clinical acquisition conditions. Unlike traditional methods that rely on subjective clinical grading, our approach integrates facial landmark detection, robust multitemporal image registration, and trimap-guided deep learning segmentation (DAM-Net). This pipeline successfully addresses the challenge of isolating fine hair structures from complex backgrounds, delivering a reproducible and operator-independent metric for assessing eyebrow density changes.

The results demonstrate that the proposed automated metric achieves high precision with an ICC of 0.971. By standardizing the Region of Interest (ROI) through adaptive geometric offsets and ensuring spatial consistency across timepoints, the methodology proves robust against natural variations in facial expression, head pose, and lighting conditions. Consequently, this framework offers a level of granularity and sensitivity that far exceeds current ordinal grading scales, enabling the detection of subtle, sub-clinical changes in hair density that might otherwise go unnoticed in initial stages of treatment.

While this pilot study was conducted on a limited cohort and was not intended to evaluate the clinical efficacy of cryotherapy itself, the results support the feasibility of this approach and establish it as a methodological foundation for periocular hair loss quantification.

The ability to generate continuous, quantitative data provides a powerful endpoint for comparing preventive interventions, allowing researchers to measure not only the presence or absence of hair loss, but the rate and magnitude of preservation. The presented framework suggests a scalable and transferable approach that could be adapted for large-scale and multicentre clinical trials following further evaluation of its inter-session and cross-device performance. Future work will focus on integrating this pipeline into accessible mobile health platforms to facilitate remote patient monitoring. Beyond the scope of CIA, this methodology holds promise for broader applications in oncodermatology and trichology, supporting the standardization of objective image-based endpoints in supportive cancer care and quality-of-life assessment.

## Data Availability

The code developed and used for the automated computer vision pipeline is available in the TIDOP-USAL GitHub repository: [https://github.com/TIDOP-USAL/crioma\_madarosis-quantification](https:/www.google.com/search?q=https:/github.com/TIDOP-USAL/crioma_madarosis-quantification) .The datasets generated and/or analysed during the current study are not publicly available due to participant privacy and confidentiality restrictions, as they contain sensitive information that could compromise the anonymity of the subjects, but are available from the corresponding author on reasonable request.
